# Effectiveness of telerehabilitation for adults with neurological conditions in low and middle income countries: A systematic review

**DOI:** 10.1371/journal.pdig.0000911

**Published:** 2025-07-07

**Authors:** Eric Nkansah Opoku, Lorna Paul, Derrick Antwi, Katie Thomson, Shadrack Osei Asibey, Marian C. Brady, Frederike van Wijck, Fred Stephen Sarfo

**Affiliations:** 1 Research Centre for Health, Glasgow Caledonian University, Glasgow, Scotland; 2 Kwame Nkrumah University of Science and Technology, Kumasi, Ghana; The University of Hong Kong, HONG KONG

## Abstract

Neurological conditions including stroke, multiple sclerosis, and Parkinson’s significantly contribute to disability and mortality globally. A significant proportion of these cases are found in Low and Middle Income Countries (LMICs). Telerehabilitation has emerged as a promising approach to overcome the geographical, financial, and logistical barriers to rehabilitation in LMICs. However, to date, no review has assessed the effectiveness of telerehabilitation in LMICs. This systematic review aims to evaluate the effectiveness of telerehabilitation for adults with neurological conditions in LMICs. A systematic search of databases (PubMed, EMBASE, CINAHL, MEDLINE, Cochrane central registry for clinical trials, and the World Health Organization (WHO) Global Health Library) was conducted to identify relevant studies published from 1^st^ January 1990–20^th^ April 2024. To accurately capture relevant studies, search terms were closely aligned with PICO elements of the review question. PRISMA and AMSTAR guidelines were used to guide the conduct of this review which only included clinical trials. Joanna Briggs Institute (JBI) critical appraisal tools were used to assess the methodological quality of included studies.Out of 430 identified studies, 16 met the inclusion criteria. There was notable heterogeneity in telerehabilitation content, approaches, dose, delivery methods and follow-up periods. Given the heterogeneity, a narrative analysis was conducted. Findings from the included studies suggest that telerehabilitation can lead to similar or superior outcomes compared to conventional rehabilitation. However, only a third of the included studies incorporated follow-up assessments, and among those, sustained benefits were observed in only a few outcomes. The lack of long-term follow-up data makes it difficult to draw conclusions about the sustained effectiveness of telerehabilitation.This systematic review indicates a promising potential for telerehabilitation to enhance outcomes for adults with neurological conditions living in LMICs. However, the lack of long-term follow-up data limits understanding of sustained benefits. High-quality, methodologically rigorous research with extended follow-up is needed to determine the long-term effectiveness of telerehabilitation. Establishing this evidence base is critical for integrating telerehabilitation into healthcare strategies and policy.

## Author summary

This systematic review evaluates the effectiveness of telerehabilitation for adults with neurological conditions in Low and Middle Income Countries (LMICs). Despite the significant burden of neurological conditions in LMICs, there is a lack of evidence regarding the effectiveness of telerehabilitation in this context. Through a rigorous search of key databases and adherence to PRISMA and AMSTAR guidelines, 16 studies were identified for inclusion. The review highlights considerable heterogeneity in telerehabilitation interventions, yet suggests that such programmes could yield comparable or even superior outcomes to conventional rehabilitation methods. However, it emphasises the necessity for further high-quality research with long-term follow-up to conclusively establish the effectiveness of telerehabilitation across LMIC settings over time. This evidence is crucial for informing healthcare strategies and policy decisions regarding the integration of telerehabilitation into neurological care in LMICs.

## 1. Introduction

Neurological conditions, such as stroke, multiple sclerosis and Parkinson’s disease are among the leading causes of disability and death, affecting millions of people worldwide with a substantial burden in Low and Middle Income Countries (LMICs) [1,2]. These conditions significantly impact the quality of life of individuals and their families, as well as the economic development of countries affected [1]. Despite the high burden of neurological conditions in LMICs, access to rehabilitation services is limited [3]. The WHO reports that there is a global shortage of healthcare professionals, especially rehabilitation professionals, with the shortage being particularly pronounced in LMICs [3]. Additionally, the infrastructure needed for rehabilitation services, such as equipment and facilities, is often inadequate or non-existent in LMICs [4]. The WHO Rehabilitation 2030 Call for Action reiterates these challenges, emphasising the need for integrating comprehensive rehabilitation services into health systems globally, and for people in need to have access to rehabilitation without being exposed to financial hardship. It emphasises the urgency of improving rehabilitation services, with a focus on workforce development, infrastructure strengthening, and policy support to guarantee equitable access and improved health outcomes [5].

Telerehabilitation has emerged as a promising approach to overcome the geographical, financial, and logistical barriers to rehabilitation in LMICs [1,4,6–8]. It involves the use of communication systems (such as telephone, videoconferencing, mobile applications, and other digital tools) by rehabilitation professionals to provide rehabilitation services remotely for people with disabilities and their families [9]. Telerehabilitation has the potential to increase access to rehabilitation services in remote and underserved areas, reduce healthcare costs, and improve patient satisfaction [1,2,10]. Moreover, it may enable earlier initiation of rehabilitation, which is crucial for maximising recovery and preventing secondary complications for adults with neurological conditions [11].

Several systematic reviews have investigated the effectiveness of telerehabilitation in adults with neurological conditions. For example, Sarfo et al. [12] examined the effectiveness of telerehabilitation in stroke survivors and found that this produced better or equal effectiveness in improving motor function, higher cortical function and mood compared with conventional rehabilitation. Similarly, Özden [13] examined the effect of a mobile-phone based rehabilitation in persons with Parkinson’s Disease and found it to be beneficial in improving different aspects of participants’ quality of life and adherence to treatment protocols.

Despite the potential benefits of telerehabilitation, its effectiveness in adults with neurological conditions in LMICs has not been well-established. Most studies included in existing reviews on this topic have been conducted in High-Income Countries (HICs), which may have different healthcare systems, social and cultural norms, and technological infrastructure compared to LMICs. There is therefore a need to evaluate the effectiveness of telerehabilitation in LMICs settings specifically. Recent figures suggest that the telecommunication infrastructure to support telerehabilitation is improving in LMICs - although not consistently - and the number of people with smart phones is significantly increasing significantly [6]. Therefore, it is timely to investigate if telerehabilitation may have the potential to improve clinical outcomes in people with neurological conditions in LMICs.

Solomon et al [14] conducted a systematic review into the effectiveness of telerehabilitation interventions specifically for individuals with spinal cord injury in LMICs and found a significant improvement in quality of life, functional ability, reduced sense of isolation and improved satisfaction scores. However, as their study included only three randomised controlled trials, all of which were of low to moderate quality, they concluded that although there is potential patient benefit, there is insufficient evidence to recommend telerehabilitation as an intervention to treat spinal cord injury in LMICs.

The current systematic review included a broader range of common neurological conditions aiming to evaluate the effectiveness of telerehabilitation for adults in LMICs. The research question this review aims to address is: ‘What are the effects of telerehabilitation, compared to a control intervention, on any outcome in people with a disabling disorder of the central nervous system in LMIC?’ By expanding the scope beyond spinal cord injury to include all conditions of the central nervous system (CNS), our review will provide a more comprehensive understanding of the effectiveness of telerehabilitation interventions in adults with neurological conditions in these settings. This review will provide valuable insights for healthcare providers, policymakers, and researchers working to improve access to rehabilitation services in these regions.

## 2. Methods

The Preferred Reporting Items for Systematic Reviews and Meta-analysis (PRISMA) guidelines [15] and A Measurement Tool to Assess systematic Reviews (AMSTAR) [16] guidelines were used to guide this systematic review with the protocol registered in PROSPERO (registration number: CRD42023444153).

### 2.1 Literature search strategy

We identified relevant studies by searching the following electronic databases: PubMed, EMBASE, CINAHL, MEDLINE, the Cochrane Central Register of Clinical Trials, and the World Health Organization (WHO) Global Health Library (African Index Medicus, Index Medicus for the Eastern Mediterranean Region, Index Medicus for the South-East Asia Region, Latin American and Caribbean Health Science Information database, and the Western Pacific Region Index Medicus). A comprehensive search was developed using MeSH and keywords for “telerehabilitation” AND “neurological condition” AND “low and middle income country” (See S1 Appendix for full search strategy). The search was restricted to studies published from January 1, 1990 to April 20, 2024, and we applied a consistent classification for Low and Middle Income Countries based on the World Bank’s criteria at the start of this search window, without revising designations for countries that transitioned in income status during this period [17]. Additionally, reference lists of both included primary studies (after full text screening) and relevant literature reviews were searched for additional studies. No language restrictions were used.

### 2.2 Inclusion and exclusion criteria

Selection criteria were based on the PICO elements of the research question for this review. Therefore, we included studies of adults with neurological conditions in LMICs who received telerehabilitation as an intervention for the management of their condition. Telerehabilitation was defined as the use of telecommunication methods to remotely provide rehabilitation services for patients and their families [9]. All telerehabilitation approaches such as telephone, internet, video and audio conferencing, robot assisted rehabilitation, and virtual and augmented reality therapy with remote interaction or monitoring were included. Comparator groups were conventional rehabilitation or inactive control (no rehabilitation). As this review aimed to evaluate intervention effectiveness, only clinical trials (i.e., randomised controlled trials, quasi-experimental studies and pilot trials) were included. Pilot trials were included for their role in evaluating the preliminary effectiveness of interventions which is crucial in the evolving field of telerehabilitation.

### 2.3 Study selection and data extraction

Studies identified using the search strategy were uploaded into Rayyan systematic review software [18]. Following removal of duplicates, title and abstracts of studies were screened. Full texts of studies were assessed using the inclusion and exclusion criteria. Two reviewers (ENO and DA) performed the screening independently at every stage. Conflict was resolved by discussion until consensus was reached between the two reviewers and in cases where consensus was not reached, the opinion of a third reviewer (LP) was sought. Relevant data from included studies were extracted into a Microsoft Excel Spreadsheet by three independent reviewers (ENO, KT and LP). Specific data extracted included author names and publication year, country of study, study aim/objectives, study design, population (number of participants, age, sex), interventions implemented (content, dose, mode of delivery), outcome measures, and results regarding effectiveness.

### 2.4 Quality assessment and data analysis

Critical appraisal of included studies was performed by two independent reviewers (LP and KT) for methodological quality using standardised critical appraisal instruments from JBI SUMARI for RCTs and quasi-experimental studies [19]. Conflict was resolved by consensus or by consulting a third reviewer (ENO). A meta-analysis of the included studies was planned but could not be undertaken due to the generalised heterogeneity of the included studies. A narrative synthesis is therefore presented.

### 2.5 Funding sources

This systematic review is part of a bigger telerehabilitation project funded by the Academy of Medical Sciences Global Challenges Research Network Grant (https://acmedsci.ac.uk/grants-and-schemes/grant-schemes/gcrf-networking-grants) (Ref GCRFNGR5\1278 to all authors of this systemative review). The funders had no role in the study design, data collection and analysis, decision to publish, or preparation of the manuscript.

## 3. Results

### 3.1 Search results

**Fig 1:**
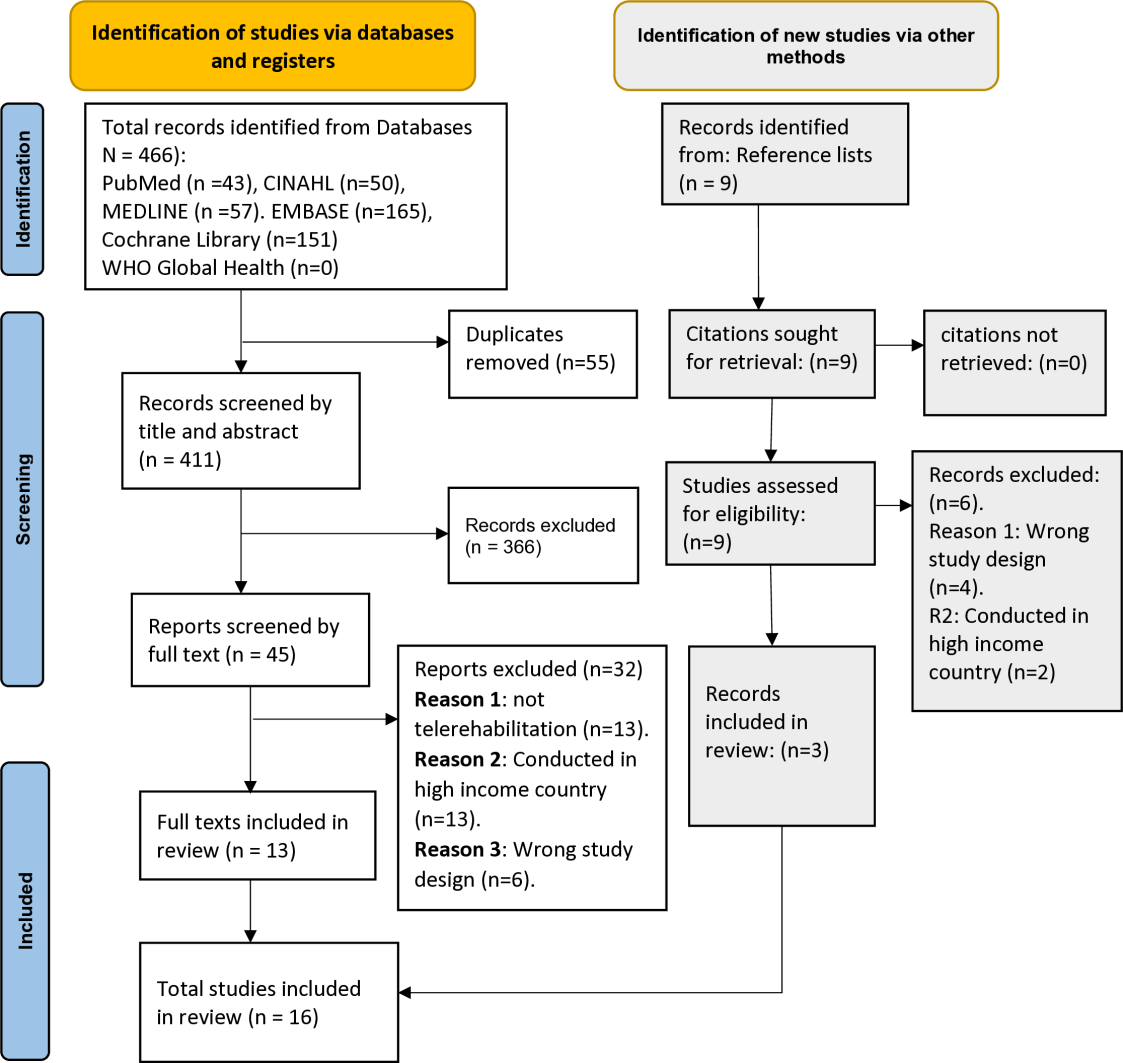
The PRISMA flowchart of the review.

The search yielded a total of 466 studies from the following databases: PubMed (n = 43); CINAHL (n = 50); MEDLINE (n = 57); EMBASE (n = 165); Cochrane Library (n = 151) and WHO Global Health (n = 0). An additional 9 studies were identified through searching reference lists. Following duplicate removal and the screening processes, 16 studies were eligible for inclusion and relevant data were extracted and analysed to form this systematic review (Fig 1).

### 3.2 Methodological quality

All 16 studies included in this review were critically appraised for methodological quality as appropriate to their study design (Table 1 and Table 2). Fifteen studies were RCTs [20–34] and one was a Quasi-experimental study. The RCT studies demonstrated generally sound methodologies but varied in specific domains of risk. In terms of selection and allocation, all studies used robust randomisation methods (Q1) and most reported adequate allocation concealment (Q2) except for three studies which were unclear [26,29,31]. All studies except one [23] had similar treatment groups at the baseline (Q3). Administration of the telerehabilitation intervention varied in blinding (Q4, Q5). As expected for this type of intervention, nearly all studies were unable to blind participants or those delivering the intervention, although most studies provided comparable treatment (Q6). Outcome assessment was largely consistent and reliable (Q8, Q9), but many studies did not clearly report whether outcome assessors were blinded (Q7), introducing a risk of detection bias. Participant retention (Q10) was largely reported, but Redzuan et al. [23] did not account for group follow-up differences, raising concerns about attrition bias. For statistical conclusion validity, all of the RCT studies employed appropriate statistical analyses (Q12) and provided sufficient detail on the trial design (Q13). The quasi-experimental study [35] demonstrated areas of strength including a clear cause-and-effect relationship (Q1) with comparable participant groups (Q3). It was however unclear whether outcomes of participants included in any comparison were measured consistently (Q7).

**Table 1:** Critical appraisal results of RCTs [19].

RCT Studies	Internal validity Bias related to:												
	Selection and allocation			Administration of intervention			Assessment, detection, and measurement of the outcome			Participant retention	Statistical conclusion validity		
	Q1	Q2	Q3	Q4	Q5	Q6	Q7	Q8	Q9	Q10	Q11	Q12	Q13
Chaiyawat & Kulkantrakorn [33]	Y	Y	Y	N	N	Y	N	Y	?	Y	Y	Y	Y
Chen et al [32]	Y	Y	Y	N	N	Y	Y	Y	Y	Y	Y	Y	Y
Dogan et al [34]	Y	Y	Y	?	?	Y	Y	Y	Y	Y	Y	Y	Y
Eldemir et al [31]	Y	?	Y	?	?	Y	Y	Y	Y	Y	Y	Y	Y
Guo et al [30]	Y	Y	Y	N	?	Y	Y	Y	Y	Y	Y	Y	Y
Hashemi et al [29]	Y	?	Y	Y	N	Y	Y	Y	Y	Y	Y	Y	Y
Kahraman [28]	Y	Y	Y	?	?	Y	Y	Y	Y	Y	Y	Y	Y
Khoja et al [27]	Y	Y	Y	N	N	Y	Y	?	Y	Y	Y	Y	Y
Lin et al [26]	Y	?	Y	N	N	Y	Y	Y	Y	Y	Y	Y	Y
Liu et al [25]	Y	Y	Y	N	NA	Y	Y	Y	Y	Y	Y	Y	Y
Liu et al [24]	Y	Y	Y	N	N	Y	Y	Y	Y	Y	Y	Y	Y
Redzuan et al [23]	Y	N	N	N	N	N	Y	Y	?	Y	Y	Y	Y
Wan et al [22]	Y	Y	Y	N	N	Y	Y	Y	Y	Y	Y	Y	Y
Wu et al [21]	Y	Y	Y	N	N	Y	Y	Y	Y	Y	Y	Y	Y
Zarei et al [20]	Y	Y	Y	N	?	Y	?	Y	Y	Y	Y	Y	Y

Y: Yes, N: No,?: Unclear, NA: Not Applicable JBI Critical appraisal checklist questions for RCTs:

Q1: Was true randomization used for assignment of participants to treatment groups? Q2: Was allocation to treatment groups concealed? Q3: Were treatment groups similar at the baseline? Q4: Were participants blind to treatment assignment? Q5: Were those delivering treatment blind to treatment assignment? Q6: Were treatment groups treated identically other than the intervention of interest? Q7: Were outcomes assessors blind to treatment assignment? Q8: Were outcomes measured in the same way for treatment groups? Q9: Were outcomes measured in a reliable way? Q10: Was follow up complete and if not, were differences between groups in terms of their follow up adequately described and analysed? Q11: Were participants analysed in the groups to which they were randomized? Q12: Was appropriate statistical analysis used? Q13: Was the trial design appropriate, and any deviations from the standard RCT design (individual randomization, parallel groups) accounted for in the conduct and analysis of the trial?

**Table 2:** Critical appraisal results of Quasi-experimental studies [19].

Quasi-Experimental Studies	Q1	Q2	Q3	Q4	Q5	Q6	Q7	Q8	Q9
Kazemi et al [35]	Y	Y	Y	Y	Y	?	?	Y	Y

Y: Yes, N: No,?: Unclear.

JBI Critical appraisal checklist questions for Quasi-experimental studies [19]: Q1: Is it clear in the study what is the ‘cause’ and what is the ‘effect’ (i.e., there is no confusion about which variable comes first)? Q2: Were the participants included in any comparisons similar? Q3: Were the participants included in any comparisons receiving similar treatment/care, other than the exposure or intervention of interest? Q4: Was there a control group? Q5: Were there multiple measurements of the outcome both pre and post the intervention/exposure? Q6: Was follow up complete and if not, were differences between groups in terms of their follow up adequately described and analysed? Q7: Were the outcomes of participants included in any comparisons measured in the same way? Q8: Were outcomes measured in a reliable way? Q9: Was appropriate statistical analysis used?

### 3.3 Study characteristics

The main characteristics of the included studies (authors, study objectives, participant information, and telerehabilitation/comparator intervention components) are summarised in Table 3. The studies were conducted in China (n = 7), Iran (n = 3), Turkey (n = 3), Thailand (n = 1), Pakistan (n = 1), and Malaysia (n = 1). Overall, 12 of the studies were conducted in Upper Middle Income Countries [21–26,28,30–34], and four studies in Lower Middle Income Countries [20,27,29,35].

**Table 3:** Summary of included studies.

Author (Year)/Study design/ Country	Study Aim/Objective	Study participants	Telerehabilitation Intervention (Intervention Group (IG))	Comparator Intervention Control Group (CG))
Chaiyawat & Kulkantrakorn (2012). [33] RCT Thailand	To develop and examine the effectiveness of an individual 6-month home rehabilitation program on disability and quality of life for participants with ischemic stroke at 2 years post stroke.	60 participants with stroke due to middle cerebral artery infarction. Intervention Group (IG)= 30 (M = 30, F = 0); Age (SD)= 67 (±10) yrs. Control Group (CG) = 30 (M = 30, F = 0); Age (SD)= 66 (±11) yrs.	Home-based physical mobility exercises provided once a month for 6-months lasting approximately 1 hour, followed by activities of daily living training programme using audio visual instructional materials. Follow up conducted every month for 6months	Participants received usual care which included pre-discharge instructions for home rehabilitation, as well as outpatient rehabilitation. No follow up visits.
Chen et al, 2017. [32] RCT China	To determine whether or not home-based remotely supervised telerehabilitation is better than conventional outpatient rehabilitation on recovery of physical function for stroke survivors with hemiplegia.	54 participants with ischemic or hemorrhagic stroke, aged 35–85 yrs. IG: n = 27 (M = 18; F = 9); Age (SD): 66.52 (±12.08) yrs. CG: n = 27 (M = 15; F = 12); Age (SD)= 66.15 (±12.33) yrs.	IG: Remote supervision of physical exercises and electromyography-triggered neuromuscular stimulation (ETNS) by live video conferencing. Physical exercises were conducted for 1 hour, twice in a working day for 12 weeks. ETNS was conducted for 20 minutes, twice in a working day for 12 weeks.	Participants received outpatient rehabilitation. Therapeutic strategy was the same as the intervention group, but was carried out face-to-face by trained therapists rather than by telerehabilitation.
Dogan et al (2023) [34] RCT Turkey	To investigate the effects of two different technology-supported rehabilitation approaches: mobile application based telerehabilitation (TR) and virtual reality supported task-oriented circuit therapy groups (V-TOCT) on the upper limb (UL), trunk function, and functional activity kinematics in patients with Multiple Sclerosis (MS).	34 participants with MS. IG: n = 17 (M = 2; F = 15); Age (SD)= 36 (±8.19) yrs. CG: n = 17 (M = 6; F = 11); Age (SD): 38.76 (±5.53) yrs.	Mobile application based telerehabilitation intervention consisted of a home-based exercise program that included balance, strengthening, coordination, and stretching exercises. Participants received 1-hour sessions, 3 times per week, for 8 weeks.	Virtual reality supported task-oriented circuit therapy groups (V-TOCT) consisted of computer games that imitated daily tasks, providing unilateral, bilateral, and bimanual UL training. Participants received 1-hour sessions, 3 times per week, for 8 weeks.
Eldemir et al (2023) [31] RCT Turkey	To investigate the effectiveness of task-oriented circuit training-based telerehabilitation (TOCT-TR) on the upper extremity motor function in People with Parkinson’s Disease (PD).	30 participants with PD IG: n = 15 (M = 10; F = 5); Age (SD)= 57.87 (± 9.79) yrs. CG: n = 15 (M = 9; F = 6); Age (SD)= 61.40 (± 7.29) yrs.	Training consisted of a videoconferencing work station involving 15 different manual dexterity and upper extremity exercises. Participants received interventions for 60 mins, 3 days per week for 6 weeks.	Home based exercise intervention consisting of exercises aimed at improving balance, gait, and mobility. Participants were given a booklet explaining the exercises with pictures, a diary to complete and telephone follow-up calls twice a week.
Guo et al (2023) [30] RCT China	To evaluate the effectiveness and safety of a remote rehabilitation training system for non-physician-supervised motor rehabilitation training of patients with stroke	120 inpatients with stroke IG: n = 60 (M = 43 F = 17); Age (SD)= 56.25 (± 9.83) yrs. CG: n = 60 (M = 43; F = 17); Age (SD)=55.82 (± 9.68) yrs.	Remote rehabilitation training system for rehabilitation and routine clinical physical therapy. Training comprised of coordinated movement training of the upper extremity, hand, and lower extremity. Participants trained at least 10 times a week, for a total of 3 weeks.	Routine occupational therapy (OT) and physical therapy training. Participants trained no less than 10 times a week, for a total of 3 weeks.
Hashemi et al., 2022. [29] RCT Iran	To investigate the effects of supervised and non-supervised Upper Limb Virtual Reality Exercises (ULVRE) on UL sensory-motor function in participants with idiopathic PD.	45 participants with idiopathic PD Supervised ULVRE: n = 15 (M = 9; F = 6); Age (SD)=54.80 (± 10.51) yrs. Non-supervised ULVRE: n = 15 (M = 8; F = 7); Age (SD)=58.64 (± 8.69) yrs. Control Group n = 15 (M = 12; F = 3); Age (SD)=61.07 (± 7.01) yrs.	Two intervention groups: In both the supervised and non-supervised ULVRE group, participants received a range of virtual reality exercises in addition to conventional rehabilitation exercises for 8 weeks. Supervised ULVRE took place in an outpatient rehabilitation centre, non-supervised ULVRE occurred in participants’ homes. Interventions included 24 sessions, 3 times per week for 75mins per session.	Conventional rehabilitation of physical exercises and activities of daily living training. There were 24 sessions, 3 times per week, 90 min per session.
Kahraman (2020) [28] RCT Turkey	To investigate the effects of telerehabilitation-based motor imagery training (Tele-MIT) on gait, balance and cognitive and psychosocial outcomes in people with Multiple Sclerosis.	55 participants IG: n = 20 (M = 4; F = 16); Average Age = 34.5yrs. CG: n = 15 (M = 1; F = 14); Average Age = 36yrs. Healthy group: n = 20 (M = 6; F = 14); Average Age = 31yrs.	Relaxation exercises and multisensory environmental information provided using auditory, visual, tactile, and olfactory cues. Intervention sessions of 20–30 mins, twice per week for 8 weeks.	Multiple Sclerosis control group: no intervention Healthy control group: no intervention
Kazemi et al., 2022. [35] Quasi- experimental with random allocation Iran	To evaluate the effects of continuous care model using a smartphone application on adherence to treatment and self-efficacy among participants with relapsing-remitting Multiple Sclerosis	72 participants (males = 24; females = 48) Age range = 18–45 yrs. IG: n = 36 CG: n = 36	Continuous care model, via an interactive educational multimedia smartphone application for 4 months. The care comprised interactive educational multimedia content on MS and self-management of MS. Follow-up at 2 months post intervention end.	Control group - received routine care only
Khoja et al, 2018. [27] RCT Pakistan	The effectiveness of a customized mHealth software program that showing short 5-minute movies on post-stroke survival, medication adherence, response to emergencies, home-based rehabilitation and dysphagia exercises for survivor-caregiver dyads.	310 stroke survivor – caregiver dyads with first stroke (both ischemic or hemorrhagic). IG: n = 155 (M = 108; F = 47); Age (SD)=60.6 (± 12.0) yrs. CG: n = 155 (M = 101; F = 54); Age (SD)=59.7 (±14.3) yrs.	Received standard care and video-based health education intervention delivered through short videos that focused on emergency preparedness, secondary stroke prevention including rehabilitation techniques (physical activity, exercise and diet modification). Videos were shown to participants with stroke and their caregivers in sessions at the time of admission, before discharge, at first- and third-month post discharge. Outpatient follow-up visit 1^st^, 3^rd^, 6^th^, 9^th^ and 12^th^ months.	Standard care only, including pre-discharge education and counseling according to defined protocols of the center.
Lin et al., 2014. [26] RCT China	To evaluate the effect of a bidirectional and multi-user telerehabilitation system on balance and satisfaction in participants with chronic stroke living in long-term care facilities.	24 participants with chronic stroke living in long-term care facilities. IG: n = 12 (M = 10; F = 2); Age (SD)=74.6 (±2.3) yrs. CG: n = 12 (M = 7; F = 5); Age (SD)= 75.6 (±3.4) yrs.	Balance and functional activity exercises via a telerehabilitation programme 3 times per week for 4 weeks for approximately 50 minutes per session.	Conventional therapy consisting of physical exercises, three times per week, for 4 weeks?? for 50 minutes per session
Liu et al, 2021. [25] RCT China	To evaluate the effects of app-based transitional care on self-efficacy and quality of life of participants with SCI.	98 participants with SCI living at home following discharge. IG: n = 49 (M = 41; F = 8); Age (SD)=40.37 (±12.18) yrs. CG: n = 49 (M = 40; F = 9; Age (SD)=43.06 (±12.06) yrs.	Received remote transitional care program via mobile app which had four core functions: remote assessment, health education, interdisciplinary referral, and patient interaction, at weeks 2, 4, 6, 8, and 12 following discharge. Intervention lasted 12 weeks with follow up after 24 weeks.	No post discharge transitional care services. Post-discharge telephone follow-up at week 12, and after 24 weeks.
Liu et al., 2023. [24] RCT China	To assess the effect of a self-management intervention delivered by mobile application for depression among ommunity-dwelling individuals with spinal cord injury	98 Community-dwelling participants. IG: n = 49 (M = 41; F = 8); Age (SD): 40.37 (±12.18) yrs. CG: n = 49 (M = 40; F = 9); Age (SD)= 43.06 (±12.06) yrs.	Received usual care (consisting of health education, functional exercise training and home modifications), and five sessions of self-management training covering caregivers’ ability, body structure, body function, activity and participation, and contextual factors, provided by a multidisciplinary team via mobile application at week 2, 4, 6, 8 and 12 after discharge from hospital. Intervention lasted 12 weeks and follow up after another 12 weeks.	Usual care (consisting of health education, functional exercise training and home modifications), and follow-up telephone counselling provided by nurses at week 12 following discharge from hospital.
Redzuan et al, (2012). [23] RCT Malaysia	To evaluate the effectiveness of an intervention using video to deliver therapy at home for participants with stroke.	90 participants with stroke. IG: n = 44 (M = 21; F = 23); Age (SD)=63.7 (±12) yrs. CG: n = 46 (M = 31; F = 15); Age (SD)=59.4 (± 11) yrs.	A combination of at-home rehabilitation including moving and handling, mobility and activities of daily living exercises guided by a digital videodisk containing therapy techniques and twice-monthly outpatient follow-up for 3 months. Participants were encouraged to complete the exercises along with the video as often as possible. Intervention duration: 3 months	Conventional therapy via outpatient appointments and education on how to perform therapy at home.
Wan et al., 2016. [22] RCT China	To evaluate the effectiveness of a guideline-based, goal-setting telephone follow-up program for participants with ischemic stroke.	91 participants with Ischemic stroke IG: n = 46 (M = 30; F = 16); Age Range = 35–86yrs. CG: n = 45 (M = 27; F = 18); Age Range = 35–86yrs.	Pre-discharge education on stroke and risk factors and 3 goal-setting sessions at 1 week, 1 month and 3 months after discharge, each lasting 15–20 minutes, conducted by phone.	Usual care education on stroke and risk factors, and outpatient appointments.
Wu et al., (2020). [21] RCT China	To explore the feasibility and effectiveness of a collaborative care model-based telerehabilitation exercise training for participants with acute stroke.	64 adults with acute stroke aged 18–80 years. IG: n = 32 (M = 19; F = 11); Age (SD)= 56.73 (± 11.85) yrs. CG: n = 32 (M = 17; F = 14); Age = 59.10 ± 8.60yrs	After discharge, received personalised home remote rehabilitation involving health education, physical and training in activities of daily living at varied doses, based on a collaborative care model, twice a week. Intervention duration was 12 weeks.	After discharge, control group received routine rehabilitation and nursing guidance conducted via telephone follow-up once a week.
Zarei et al 2020. [20] RCT Iran	To assess the effectiveness of a mobile phone-based educational intervention targeting sex and marital life in Iranian men with spinal cord injury (SCI).	70 men diagnosed with SCI for at least 1 year previously, aged at least 18 years old, tetraplegic, married. IG: n = 35 (M = 35; F = 0), Age (SD)= 36.7, (± 39.5) yrs. CG: n = 35 (M = 35; F = 0); Age (SD)= 35.3 (± 37.7) yrs.	Mobile-based educational app designed to provide educational content on sexo marital life, installed on participants smartphones. Educational content was available for the at any time for 8 weeks; Intervention period was 8 weeks.	Participants received no intervention.

ADL – Activities of Daily Living; CG – Control Group; ETNS – Electromyography-Triggered Neuromuscular Stimulation; F – Female(s); IG – Intervention Group; M – Male(s); mHealth – Mobile Health; MIT – Motor Imagery Training; MS – Multiple Sclerosis; OT – Occupational Therapy; PD – Parkinson’s Disease; RCT – Randomised Controlled Trial; SCI – Spinal Cord Injury; SD – Standard Deviation; Tele-MIT – Telerehabilitation-Based Motor Imagery Training; TOCT-TR – Task-Oriented Circuit Training–Based Telerehabilitation; TR – Telerehabilitation; UL – Upper Limb; ULVRE – Upper Limb Virtual Reality Exercises; V-TOCT – Virtual Reality–Supported Task-Oriented Circuit Therapy; VR – Virtual Reality; yrs – Years.

### 3.4 Participant characteristics

Across all reviewed studies, a total of 1315 participants were included, with various neurological conditions. The number of participants in any single study varied from 24 participants [26] to 310 participants [27], with overall a mix of males (875 participants) and females (439 participants) with an age range from 18 to 85 years. Stroke was the predominant condition studied, involving a total of 813 participants across eight studies [21–23,26,27,30,32,33]. Spinal cord injury was studied across three studies with a total of 266 participants [20,24,25], Multiple Sclerosis was studied across three studies with a total of 161 participants [28,34,35] and idiopathic Parkinson’s Disease was studied across two studies including a total of 75 participants [29,31].

### 3.4 Telerehabilitation interventions

The content, dose and delivery methods of the telerehabilitation interventions varied widely within the studies included (Table 3). Nine of the interventions were based on functional exercises and activities of daily living tasks [23,26,27,29–34] and six were health education oriented [20,22,24,25,27,35]. Khoja et al. [27] adopted multiple intervention approaches including health education, functional exercises, and diet modification. Other interventions focused on diet modification [27], drug administration [30] depression management [24] and sex and marital life [20]. The interventions were administered through a range of methods including the telephone [22], mobile applications [20,24,25,34,35], synchronous video conferencing [21,26,28,31,32], asynchronous videos [23,27,33], virtual reality [29] and computer-based video gaming [30]. Intervention duration ranged from 2 months [20] to 24 months [33], with frequency varying from twice per working day for 12 weeks [32] to twice monthly for 3 months [23]. The total number for sessions ranged from 6 [23] to 24 [32], with session duration ranging from 15-20 minutes [22] to 75 minutes per session [29]. Five of the 16 included studies conducted follow-ups, which ranged from 8 weeks to 24 months post intervention [25,29,30,32,35].

### 3.5 Comparator interventions

The control group interventions also varied across the studies included, but generally involved a range of non-telerehabilitation interventions, including pre-discharge instructions, outpatient rehabilitation, and conventional therapy. Common across several studies was the provision of usual care including pre-discharge education, counselling, and routine rehabilitation [21,27,30,31,33,35]. Some studies highlighted no intervention or inactive educational content for control groups [20,28], while others focused on conventional rehabilitation through outpatient appointments, emphasising physical exercises and training in activities of daily living [22,23,26,29]. Two studies incorporated post-discharge strategies as control interventions, such as post-discharge telephone contacts without transitional care services [24,25], aiming to support patients after hospital discharge (Table 4).

**Table 4:** Summary of results.

Trial/Study design/ Country	Telerehabilitation method	Outcome measures and measurement time points	Results
Chaiyawat & Kulkantrakorn (2012). [33] RCT Thailand	Video-based (asynchronous)	Barthel index (BI) Utility index (EQ-5D) modified Rankin Scale (mRS) Outcomes measure before and after 2 years Intervention period	**Post intervention results at 2 years:** **BI:,** A significant improvement in IG compared with CG: 97.2 ± 2.8 **vs**. 76.4 ± 9.4, p < 0.001 **EQ-5D:** A significant improvement in the IG compared with CG: 0.9 ± 0.02 **vs** 0.7 ± 0.04, p = 0.03 **mRS:** A Significant improvement in the IG compared with the CG after 2 years: Min or no disability, n(%) = 28(93.3%) vs 9(32.1%); Moderate disability, n(%) = 2(6.7%) vs 19(67.9%). p = 0.02
Chen et al, 2017. [32] RCT China	Video conferencing (synchronous)	modified Barthel Index (mBI) Berg Balance Scale (BBS) modified Rankin Scale (mRS) Caregiver Strain Index (CSI) Root mean square (RMS) of EMG signals of ex-tensor carpi radialis longus and tibialis anterior muscle. Measurement at 3 time points: baseline, postintervention (12 weeks), and 12-week follow-up (24 weeks).	**Post intervention (12 wks) and follow-up (24 wks) results:** **MBI**: IG improved from 55.6 ± 12.8 to 67.3 ± 12.3 and CG from 54.3 ± 13.4 to 66.0 ± 10.8. A strong time effect was detected (*F* = 138.965, *p* < 0.001), but neither the group main effect (*F* = 0.147, *p* = 0.703) nor the group × time interaction (*F* = 0.109, *p* = 0.897) was significant. Between-group mean differences remained small and imprecise: 12 wks = 2.08 (95% CI −5.17 to 9.34); 24 wks = 1.52 (95% CI −5.01 to 8.05). **BBS**: IG improved from 33.1 ± 4.0 to 40.5 ± 4.1; CG from 31.7 ± 5.9 to 39.5 ± 5.4. Time effect: F = 907.300, p < 0.001. No group (F = 0.012, p = 0.247) or interaction (F = 1.423, p = 0.912) effects. Between-group mean differences at **12 wks** = 0.92 (95% CI −1.27 to 3.10); **24 wks** = 0.65 (95% CI −1.43 to 2.73). **mRS**: Scores improved for both groups (grade 0 or 1, from 3.7% to 57.7% in the IG; from 0% to 72% in the CG; grade 4 or 5, from 37.1% to 3.8% in the IG; from 33.3% to 0% in the CG). No significant between group difference at 12 weeks (p = 0.860) and 24 weeks (p = 0.278). **CSI** (↓ = better): Scores reduced in IG from 6.04 ± 1.72 to 3.04 ± 1.51 and in CG from 6.26 ± 2.49 to 3.33 ± 1.95. Time effect: F = 285.234, p < 0.001. No significant group (F = 0.188, p = 0.666) or interaction (F = 1.054, p = 0.356) effect. Between-group mean differences: **12 wks** = 0.41 (95% CI −0.66 to 1.49); **24 wks** = 0.43 (95% CI −0.62 to 1.49). **RMS of EMG signals of extensor carpi radialis longus**: **IG** improved from 72.2 ± 22.0 to 109.3 ± 19.8 and **CG** from 67.6 ± 15.3 to 107.2 ± 17.0. Time effect: F = 3835.666, p < 0.001; no group (F = 0.169, p = 0.688) or interaction (F = 0.104, p = 0.901) effect. Between group mean differences at **12 wks** = 2.33 (95% CI −8.86 to 13.52); **24 wks** = 2.52 (95% CI −8.53 to 13.58). **RMS of EMG signals of tibialis anterior**: IG improved from 104.7 ± 37.7 to 163.3 ± 30.3; CG 100.4 ± 28.1 to 161.1 ± 25.8. Time effect: F = 766.267, p < 0.001; no group (F = 0.001, p = 0.989) or interaction (F = 0.556, p = 0.583) effect. Between group mean differences at **12 wks** = 5.08 (95% CI −11.11 to 21.28); **24 wks** = 4.26 (95% CI −12.55 to 21.07).
Dogan et al (2023) [34] RCT Turkey	Mobile application-based	Primary measures: -The Trunk Impairment Scale (TIS) -The ‘kinetic function sub-parameter’ of the International Cooperative Ataxia Rating Scale (K-ICARS) Secondary measures: -The ABILHAND hand Function Questionnaire-Stroke -Minnesota Manual Dexterity tests (MMDT) -Trunk and UL kinematics using inertial sensors Measurement at 2 time points: baseline and postintervention (8 weeks).	**Post intervention results:** **TIS:** A statistically significant improvement within both groups TR = 1.77 (p = 0.01); CG = 2.77 (0.01). There was no significant between group difference (p = 0.11). **K-ICARS:** A statistically significant improvement within both groups: TR = -3.85 (p = 0.045); CG = 4.94 (0.01). A significant between group difference in favour of the CG (p = 0.02). **ABILHAND (Score):** A statistically significant improvement within both groups TR = -2.32 (p = 0.04); CG = -1.99 (0.03). No significant between group difference (p = 0.98). **MMDT:** A statistically significant improvement for both groups in placing dominant hand, non-dominant hand and turning (p < 0.05). No significant between group difference for any item (p > 0.05) **Trunk and UL kinematics:** In the IG, only the Functional RoM of the trunk increased in the coronal plane and in the transversal plane (p < 0.05). For the CG, only the Functional RoM of shoulder and wrist increased in the transversal plane and the in sagittal plane (p < 0.05). There was a significant between group difference only in dynamic balance of the trunk in favour of the CG (p < 0.05).
Eldemir et al (2023) [31] RCT Turkey	Computer-based Video-conferencing	**Primary outcome measure** - Nine Hole Peg test (9-HPT) - Jebsen Hand Function Test (JHFT) - Grip strengths - Pinch strength - Unified Parkinson’s Disease Rating Scale-III (UPDRS-III). **Secondary outcome measures** - Parkinson’s Disease Rating Scale-II (UPDRS-II) - Quality of Life (QoL, PDQ-8). Measurement at 2 time points: baseline and postintervention (6 weeks).	**Post intervention results:** A significant group-by-time interactions in favour of the intervention group in the **9-HPT** (p < 0.001), the **JHFT** (p < 0.001), **grip strengths** (p < 0.001), **pinch strengths** (p ≤ 0.015), and the **UPDRS-III** (p = 0.007) in favour of the intervention group. **A significant within group improvement for both group: UPDRS-II** (p < 0.001), and **PDQ-8** (p = 0.005), but no significant between-group differences in these outcomes
Guo et al (2023) [30] RCT China	Computer-based training video based on a game	The Fugl-Meyer Motor Function Rating scale (FMMFRS) Before and after the three-week intervention	**Post intervention results:** The **total mean Fugl-Meyer score** improved by 17.56 (SD 11.65; 95% CI 14.37-20.74) in the IG and 11.98 (SD 8.46; 95% CI 9.69-14.27) in the CG. The difference between the 2 groups was statistically significant (p = 0.005) in favour of the IG. The **mean Fugl-Meyer upper extremity score** improved by 11.28 (SD 8.59; 95% CI 8.93-13.62) in the IG and 7.45 (SD 7.24; 95% CI 5.50-9.41) in the CG. The difference between the 2 groups was statistically significant (p = 0.01) in favour of the IG. The **mean Fugl-Meyer lower extremity score** improved by 6.28 (SD 5.28; 95% CI 4.84-7.72) in the IG and 4.53 (SD 4.42; 95% CI 3.33-5.72) in the CG. There was no significant difference between the 2 groups (p = 0.06).
Hashemi et al., 2022. [29] RCT Iran	Virtual reality (VR)	Hand Active Sensation Test (HAST) Wrist Position Sense Test (WPST) Box and Block Test (BBT) Nine Hole Peg Test (NHPT) Measurement was taken 8 weeks after intervention (T1) and 2 months follow up (T2)	**Post intervention and follow-up results:** **Discriminative sensory function at end of intervention**: A significant between-group improvement for HAST-weight score of both less (p = 0.02) and more affected hands (p < 0.001) in favour of the supervised VR group. A significant between group difference in HAST- total score for the more affected hand (p = 0.004) in favour of the non-supervised VR group (Tele rehab group). Other HAST scores were not significant. There was no significant between group difference at **follow-up.** **Wrist proprioception at end of intervention**: A significant improvement within the non-supervised VR group (p = 0.01) but not the control groups (p > 0.05). A significant between group difference in favour of non-supervised VR group (p = 0.007). No significant between group difference at **follow-up**. **Gross manual dexterity at end of intervention:** A significant within group improvement in grip strength for the non-supervised VR group for both less (p = 0.001) and more affected hands (p = 0.001). No significant within group improvement in the CG (p > 0.05). No significant between group difference for both more (p = 0.09) and less affected hands (p = 0.11). No significant between group difference at **follow-up.** **Fine manual dexterity at end of intervention**: A significant improvement within the non-supervised VR group for the more affected hand (p < 0.001), but not the less affected hand (p = 0.08). No significant between group difference for both the more (p = 0.07) and the less affected hand (p = 0.07). There was no significant between group difference at **follow-up**.
Kahraman (2020) [28] RCT Turkey	Video-conferencing	**Primary outcome measure:** Dynamic Gait Index (DGI) **Secondary outcome measures:** -Timed 25-Foot Walk (T25FW) -2-Minute Walk Test (2MWT) -MS Walking Scale (MSWS-12) -Timed Up and Go (TUG) -Activities-specific Balance Confidence (ABC) Scale -Modified Fatigue Impact Scale (MFIS) -Multiple Sclerosis International Quality of Life questionnaire (MusiQoL) -Symbol Digit Modalities Test (SDMT) Measurement at 2 time points: baseline and postintervention (8 weeks).	**Post intervention Results:** IG exhibited significant improvements in dynamic balance during walking (p = 0.002), walking speed (p = 0.007), perceived walking ability (p = 0.008), balance confidence (p = 0.002), most cognitive functions (p = 0.001–0.008), fatigue (p = 0.001), anxiety (p = 0.001), depression (p = 0.005) and quality of life (p = 0.002). No significant changes were observed in the control group in any of the outcome measures (p > 0.05). No significant difference between the intervention and MS control group in all study outcome measures (p > 0.05) with the exception of SDMT score (p = 0.014) in favour of IG.
Kazemi et al., 2022. [35] Quasi- experimental with random allocation Iran	Mobile application-based	Multiple Sclerosis Treatment Adherence Questionnaire (MS-TAQ) MS Self-Efficacy Scale (MSSES) Measurement at 3 time points: Assessment at baseline (T0), after intervention (4 months) and 2 months after intervention.	**Post intervention and follow-up results:** **MS-TAQ**: A significant within group difference for IG (p < 0.00001) but not CG (p = 0.539). A significant between group difference in favour of IG after intervention (p < 0.0001) and at follow up (p < 0.0001) **MSSES**: A significant within group difference for both IG (p = 0.0001) and CG (p = 0.010). A significant between group difference in favour of IG at end of intervention (p < 0.0001) and at follow up (p < 0.0001)
Khoja et al, 2018. [27] RCT Pakistan	Video-based (asynchronous)	The primary outcome measures: Morisky Medication Adherence Scale Blood pressure control Blood sugar Blood lipids Secondary outcomes: modified Rankin Scale (mRS), National Institutes of Health Stroke Scale (NIHSS) Barthel Index (BI) Measurement at 2 time points: Assessment at baseline and 12 months after intervention	**Post intervention results:** **Primary Outcomes**: None of the between group difference were statistically significant (p > 0.05). **Secondary Outcomes**: There was greater percentage of participants in the IG with minimal to no disability (71.1% vs. 59.2%), minimal neurologic deficit (50.8% vs. 45.8%), and minimal to no dependency (68.0% vs. 59.2%) than the CG, as assessed by the mRs, NIHSS and Barthel Index respectively, but between- group difference for these measures was not statistically significant (p > 0.05).
Lin et al., 2014. [26] RCT China	Computer-based Video-conferencing	Berg Balance Scale (BBS), Barthel Index (BI), telerehabilitation satisfaction of the participants. Measurement at 2 time points: Assessment at baseline and 4 weeks after intervention	**Post intervention results:** A significant improvement in the BBS within both groups (p < 0.001). No significant between-group difference (p = 0.829). A significant training effects on total Barthel Index score (p = 0.008) and the self-care component of BI (p = 0.014) but not mobility component (p = 0.088). No significant between-group difference for Barthel Index total score (p = 0.451), self-care (p = 0.543), and mobility (p = 0.557). A significant within group difference for the telerehabilitation group on Perceived Usefulness (p = 0.016) and Perceived Satisfaction of System (p = 0.049). No significant between group difference for all the items, including Perceived Usefulness (p = 0.053) and Perceived Satisfaction of System (p = 0.052)
Liu et al, 2021. [25] RCT China	Mobile application-based	Moorong Self-Efficacy Scale (MSES) Short-Form Health Survey (SF-36) Measurement at 3 time points: Assessment at baseline, at end of intervention (12 weeks) T1 and at follow up (24 weeks) T2.	**Post intervention and follow-up results:** **MSES**: Statistically significant groups x time interaction effects in favour of IG (total scores: F_2,95_ = 20.389, p < .001; social function self-efficacy: F_2,95_ = 13.445, p < .001; general self-efficacy: F_2,95_ = 16.063, p < .001; person function self-efficacy: F_2,95_ = 13.604, p < .001). No significant between-group differences at end of intervention (Total Scores: F1 = 0.125, p = 0.72; Self Function Self-efficacy: F = 0.124, p = 0.73; general self-efficacy: F1 = 1.102, p = 0.30; person function self-efficacy: F1 = 0.013, p = 0.91). The total scores at follow up significantly improved in the IG than in the CG (total scores: F1 = 8.506, p = 0.004; social function self-efficacy: F1 = 8.698, p = 0.003; general self-efficacy: F1 = 6.684, p = 0.01; person function self-efficacy: F1 = 6.684, p = 0.01). **SF-36:** Statistically significant groups x time interaction effects in favour of IG for the total scores and PCS scores at end of intervention (total scores: F_2,95_ = 6.671, p = 0.002; PCS: F_2,95_ = 24.516, p < 0.001). However, although total SF-36 score was higher for the IG (T2 = 65.36) than the CG (T2 = 58.77) at 24 weeks, only time effects were significant (F2,95 = 6.671, p = 0.002). No between-group differences (F_2,95_ = 3.059, p = 0.052)
Liu et al., 2023. [24] RCT China	Mobile application-based	Beck’s Depression Inventory (Version 2) (BDI). Outcomes taken at baseline, 12 weeks (post intervention) (T1) and follow up 24 weeks (T2).	**BDI** (mean ± SD; range) at 12 weeks [IG 15.65 ± 11.38; (1, 43)], [CG: 16.04 ± 11.19; (0, 40)]. **BDI at 24 weeks** (mean ± SD; range): [IG = 14.39 ± 12.79 (95% CI = 0, 49)]; [CG = 18.84 ± 12.68 (95% CI = 0, 45). No significant difference at T1 (B = -1.69; 95% CI = -5.43, 2.04; p = 0.374). Significant lower level of depression in IG compared to CG at T2 (B = -5.76; 95% CI = -9.97, -1.54; p = .007). Small to moderate effect size favouring the intervention at T1 (Cohen’s d = -.178) and T2 (Cohen’s d = -.535).
Redzuan et al, (2012). [23] RCT Malaysia	Video-based	**Primary outcome measure:** modified Barthel Index (mBI). **Secondary measures**: - Incidence of poststroke complications - The Caregiver Strain Index (CSI). Outcomes taken at baseline and 3 months post intervention (T1)	**Post intervention results:** **MBI score:** No significant differences between groups at 3 months (B = 8.4; 95% CI, 7.42–16.20, p = 0.030). Both groups had a significant within group increase in the MBI at 3 months (IG: B = 31.5 ± 17.08; 95% CI, 26.30 – 36.70, p < 0.001; CG: B = 25.35 ± 20.01; 95% CI, 19.40 – 31.29, p < 0.001). There was no significant difference in the **incidence of stroke-related complications** between the groups ((95% CI,.52–2.73, p = 0.68). While there was a significant difference within the intervention group, there was no significant difference in **CSI** between the two groups (95% CI,.19–1.25, p = 0.13).
Wan et al., 2016. [22] RCT China	Telephone-based	Primary outcome: Health Behaviour Scale (HBS) and its subscales Secondary outcome modified Rankin Scale (mRS) Data were collected at baseline and at 3- and 6-months post discharge	**Post intervention results:** **HBS:** Both groups showed improvements over time (p < 0.001). No significant difference between the groups (p = 0.193). No significant difference within group three months after discharge (0.390). Significantly higher medication adherence in IG than the CG six months (p = 0.016). No significant between group differences in physical activity, nutrition, low-salt diet adherence, blood pressure monitoring, smoking abstinence and unhealthy use of alcohol (p > 0.05). **mRS score:** Significant within group improvement for both group (p < 0.05). Significant difference between the two groups in favour of IG after 3 months (p < 0.001), but not after 6 months (p = 0.555).
Wu et al., (2020). [21] RCT China	Video-conferencing (synchronous)	Fugl-Meyer Motor Function Assessment (FMMFA) Berg Balance Scale (BBS) The Timed “UP & GO” test (TUG) The 6-minute walking test (6MWT) Modified Barthel Index (mBI) The Stroke-Specific Quality of Life Scale (SS-QOL) Outcomes taken at baseline and 12 weeks post intervention (T1)	**Post intervention results:** After 12 weeks, both groups had significantly improved in terms of motor function (p < 0.001) and quality of life (p < 0.001). Compared to the CG, the IG showed greater improvement in: **FMMFA:** (IG = 83.70 ± 4.44, CG = 75.29 ± 2.89; p < 0.001), **BBS:** (IG = 43.13 ± 2.32, CG = 38.29 ± 2.70; p < 0.001), **TUG** (IG = 19.50 ± 2.73, CG = 23.97 ± 3.35; p < 0.001), **6MWT** (IG = 141.63 ± 8.68, CG = 129.45 ± 7.06; p < 0.001), **MBI** (IG = 65.07 ± 4.15, CG = 60.81 ± 5.24, p < 0.001), **SS-QOL** (IG = 190.57 ± 5.09, CG = 175.90 ± 5.78; p < 0.001). Group-time interaction was significant in motor function and quality of life in favour of the intervention group at 12 weeks.
Zarei et al 2020. [20] RCT Iran	Mobile application-based	Sexo-marital life was assessed through Sexual adjustment questionnaire (SAQ), Larson’s sexual-satisfaction scale (LSAS), Spinner’s marital adjustment scale (SMAS), and ENRICH marital satisfaction scale (ENRICH-MSS). Outcomes taken at baseline and 8 weeks post intervention (T1)	**Post intervention results:** Between-group mean differences (95% CI) at 8 weeks: **SAQ:** A significant between-group difference in favour of IG after 8 weeks (8.6, CI: 7.6–9.5) (p < 0.001). **LSAS:** A significant between-group difference (16.4, CI: 14.1–18.7; p < 0.001). **SMAS:** No significant between-group difference (0.4, CI: −0.3 – 1.1, p < 0.25). **ENRICH-MSS:** A significant between-group differences in all the domains of marital satisfaction (7.3, CI: 6.4, 8.2, p < 0.001).

2MWT — 2-Minute Walk Test; 6MWT — 6-Minute Walk Test; 9-HPT/ NHPT — Nine-Hole Peg Test; ABILHAND — ABILHAND Hand Function Questionnaire-Stroke; ABC — Activities-specific Balance Confidence Scale; BBS — Berg Balance Scale; BBT — Box and Block Test; BDI — Beck’s Depression Inventory (Version II); BI — Barthel Index; CG — Control Group; CGI — Caregiver Strain Index; CI — Confidence Interval; DGI — Dynamic Gait Index; EMG — Electromyography; ENRICH-MSS — ENRICH Marital Satisfaction Scale; EQ-5D — EuroQol 5-Dimension Health-Related Quality-of-Life Index; ETNS - Electromyography-triggered Neuromuscular Stimulation; F – Female; FMMFA — Fugl-Meyer Motor Function Assessment; FMMFRS — Fugl-Meyer Motor Function Rating Scale; HAST — Hand Active Sensation Test; HBS — Health Behaviour Scale; IG — Intervention Group; JHFT — Jebsen Hand Function Test; K-ICARS — Kinetic Function sub-parameter of the International Cooperative Ataxia Rating Scale; LSAS — Larson’s Sexual Satisfaction Scale; M – Male; mBI — modified Barthel Index; MFIS — Modified Fatigue Impact Scale; mRS — Modified Rankin Scale; MMDT — Minnesota Manual Dexterity Test; MSES — Moorong Self-Efficacy Scale; MS-TAQ — Multiple Sclerosis Treatment Adherence Questionnaire; MSSES — Multiple Sclerosis Self-Efficacy Scale; MSWS-12 — Multiple Sclerosis Walking Scale-12; MusiQoL — Multiple Sclerosis International Quality of Life Questionnaire; NIHSS — National Institutes of Health Stroke Scale; PDQ-8 — Parkinson’s Disease Questionnaire-8; PCS — Physical Component Summary (SF-36); QoL — Quality of Life; RCT — Randomised Controlled Trial; RMS — Root Mean Square; RoM — Range of Motion; SAQ — Sexual Adjustment Questionnaire; SD — Standard Deviation, SDMT — Symbol Digit Modalities Test; SF-36–36-Item Short-Form Health Survey; SMAS — Spinner Marital Adjustment Scale; SS-QOL — Stroke-Specific Quality of Life Scale; T25FW — Timed 25-Foot Walk; TIS — Trunk Impairment Scale; TR — Telerehabilitation Group; TUG — Timed Up and Go; UL — Upper Limb; UPDRS-II — Unified Parkinson’s Disease Rating Scale, Part II; UPDRS-III — Unified Parkinson’s Disease Rating Scale, Part III; VR — Virtual Reality; Yrs – Years.

### 3.5 Effectiveness of telerehabilitation in adults with neurological conditions post-intervention

Due to the considerable variation in participants, experimental and control interventions, and outcome measures used, a meta-analysis was not thought to be meaningful, and therefore a narrative synthesis is presented.

#### 3.5.1 Functional independence.

Functional independence was assessed in six studies [21,23,26,27,32,33], by the modified Barthel Index [21,23,26,27,32,33] and the modified Rankin Scale [22,27,32,33]. The Barthel Index demonstrated improvement for participants who received telerehabilitation in all of the six studies. Of these, two studies reported a significant between groups difference in favour of the telerehabilitation group [21,33]. Four studies involving participants with stroke assessed functional independence using the modified Rankin Score [25,26,28,33]. In three of these [25,26,28], significant improvements were reported for both the telerehabilitation and control groups, whilst only two studies found a significant difference in favour of the telerehabilitation group [22,33].

#### 3.5.2 Motor, sensory and endurance functions.

Motor, sensory and endurance functions such as sensorimotor function and manual dexterity, balance, gait and walking endurance were assessed in eight of the studies [21,22,26,27,29,32–34], by the Fugl-Meyer Motor Function Assessment [21,30], the Hand Active Sensation Test [29], the Minnesota Manual Dexterity tests [34], Wrist Position Sense Test [29], Box and Block Test [29], the Nine Hole Peg Test [29,31] and EMG of extensor carpi radialis longus and tibialis anterior muscles [32], the Berg Balance Scale [21,26,32], Dynamic Gait Index [28], the 6-minute walking test [21]. For the studies that used the Berg Balance Scale, a significant improvement was reported within both the telerehabilitation group and comparator group in all three studies [21,26,32]. However, a significant between group difference was reported only in Wu et al [21] in favour of the telerehabilitation group. Telerehabilitation was found to significantly improve sensorimotor function [21], walking endurance [21], mobility and balance [21], wrist proprioception [24] and manual dexterity [24] with a significant between group difference reported in sensorimotor function [21,24], wrist proprioception [29], mobility and balance [21] and walking endurance [21] and in favour of telerehabilitation group.

#### 3.5.3 Quality of life.

Five studies assessed quality of life [21,25,28,31,33] by the Stroke-Specific Quality of Life Scale [21], the EuroQol-5D (EQ-5D) [33], the Multiple Sclerosis International Quality of Life questionnaire [28], the Quality of Life (QoL, PDQ-8) [31] and the 36-item Short-Form Health Survey (SF-36) [25]. In general, all five studies reported significant improvement within the telerehabilitation, as well as the comparator groups [21,25,28,31,33]. However, only two studies reported a significant between group difference, in favour of the telerehabilitation group [21,33].

#### 3.5.4 Self-efficacy.

Self-efficacy was assessed by two studies using the Multiple Sclerosis Self-Efficacy Scale (MSSES) [35] and Moorong Self-Efficacy Scale (MSES) [25]. In both studies, the total self-efficacy score improved in both the telerehabilitation group and the control group after the intervention period [25,35], with a significant between group difference in only one study [35]. Liu et al [25] recorded a significant between group difference in favour of the telerehabilitation group only after 24 weeks follow-up period.

#### 3.5.5 Other outcomes.

Other outcomes assessed included depression using the Beck Depression Inventory [24], sexo-marital life [20], medication and treatment adherence [22,27,35], and caregiver strain using the Caregiver Strain Index [23,32]. All these outcomes improved significantly within the telerehabilitation group. When compared with the control group, a significant between group difference in favour of the telerehabilitation group was reported in adherence to treatment [35], levels of depression [24] and medication adherence [22]. Telerehabilitation was also reported to significantly improve sexual adjustment, sexual satisfaction, marital satisfaction and marital adjustment of men with SCI, with a significant difference in favour of the telerehabilitation group in all except marital adjustment [20]. None of the other measures, i.e., control of blood pressure [22,27], health behaviour [22], blood sugar [27], blood lipids [27], alcohol and smoking abstinence [22], diet adherence [22], incidence of post-stroke complications [23] and fatigue management [28] demonstrated any significant difference between the intervention and the control groups.

### 3.6 Effectiveness of telerehabilitation in adults with neurological conditions at follow-up

Of the 16 studies included in this review, only five conducted follow up evaluation [24,25,29,32,35]. Time points ranged from 8 weeks [29,35] to 24 weeks [24,25]. These studies assessed outcomes such as self-efficacy [24,35], health-related quality of life [24], functional independence [32], motor and sensory skills [29,32], depression [24], care-giver strain [32] and treatment adherence [35]. In these five studies, there was no significant difference between the telerehabilitation groups and control groups for any of the outcomes except for self-efficacy [25,35] assessed using the Moorong Self-efficacy Scale (p = .004) [25] and Multiple sclerosis Self-efficacy scale (p < .0001) [35]; and treatment adherence [35] assessed using the Multiple Sclerosis Treatment Adherence Questionnaire (p < .0001).

## 4. Discussion

This systematic review aimed to examine the effectiveness of telerehabilitation for adults with neurological conditions in LMICs. A total of 16 studies involving 1,315 participants were identified, consisting of 15 RCTs and one quasi experimental study. Most of the included studies were conducted in Upper Middle-Income Countries (n = 12), with seven from China, but notably none from Lower Income Countries (LICs). Although telerehabilitation has been identified as a potential strategy to overcome rehabilitation delivery challenges in LMICs [1,5–8], the lack of clinical trials in LICs highlights a substantial gap in the current evidence base.

This gap may be attributable to various socioeconomic and infrastructural barriers that hinder both research conduct, reporting and implementation of telerehabilitation interventions in LICs. Factors such as limited internet connectivity, high costs of technology, and inadequate computer infrastructure can significantly affect the feasibility of remotely delivered interventions [36–38]. A further barrier may be the lack of qualified personnel with expertise in both clinical rehabilitation and technology, which is made worse by low digital literacy among patients and healthcare providers [36,37]. Additionally, resource constraints in healthcare systems, which must prioritise other urgent health needs, coupled with insufficient financing and legislative support for telerehabilitation innovations, further reduce opportunities to test, evaluate and ultimately adopt novel rehabilitation approaches in LICs [39].

The variability of the methodological quality of studies included in this review impacts the validity and reliability of the results of each study, necessitating cautious interpretation of the findings. Additionally, the potential for confounding factors, such as lack of dose-matching across the interventions, further complicates the assessment of the effectiveness of telerehabilitation. These methodological considerations are crucial for understanding the context within which findings of this review should be evaluated.

Overall, this systematic review suggests that telerehabilitation within LMICs post intervention, can lead to similar, or in a number ofcases, superior outcomes compared to conventional rehabilitation, particularly in relation to functional independence, sensorimotor function, mobility and balance function, walking endurance, wrist proprioception, self-efficacy, quality of life, adherence to medication and treatment, depression and dimensions of sexo-marital life [20–27,29,32,33,35]. This review adds to the growing body of evidence that telerehabilitation may be beneficial, supporting its use in LMICs and indicates that telerehabilitation may be a viable strategy to address some of the inherent challenges of conventional rehabilitation such as shortage of rehabilitation professionals, geographical limitations, and transportation issues.

Studies in this review mainly included participants with stroke and spinal cord injury, conditions that are common in LMICs and frequently lead to significant physical limitations, necessitating extensive and ongoing rehabilitation efforts [2]. Consequently, understanding the impact of telerehabilitation on these groups can have important implications for clinical practice, service design and health policy. However, the generalizability of the findings to other neurological conditions may be limited. The extensive age range of the participants (18–85 years) allows for an understanding of the applicability of telerehabilitation across diverse age demographics. However, this review could not determine if factors such as age, level of disability or socioeconomic group affected the effectiveness of telerehabilitation, due to paucity of data. A subgroup analyses based on these factors could facilitate a more comprehensive understanding of the potential effectiveness of telerehabilitation in future.

As expected, given our comprehensive inclusion criteria, there was notable heterogeneity in the populations, telerehabilitation interventions, comparator interventions, outcomes, and outcome measures across the reviewed studies. This is consistent with previous telerehabilitation systematic reviews that included studies from High Income Countries and LMICs [12,13,40]. The heterogeneity in outcomes, approaches and delivery methods meant a meta-analysis would not have been meaningful. However, it emphasises the need for a more standardised approach to designing and evaluating telerehabilitation interventions, e.g., by using core outcome sets for each condition. The predominance of exercise-based interventions in the reviewed studies highlights a focused approach on physical rehabilitation. The apparent lack of other forms of rehabilitation including cognitive, vocational and communication interventions points to a significant gap in the holistic rehabilitation needed to address the full spectrum of needs faced by individuals with neurological conditions in LMIC settings.

Motor function and functional independence were the most commonly reported outcomes. These outcomes play a critical role in an individual’s ability to perform daily tasks, and are a key target of rehabilitation for adults with neurological conditions [41]. In this review, all studies that assessed motor function and functional independence showed a significant improvement for both telerehabilitation and conventional therapy groups [21,23,26,27,32,33].. Some studies found a significant between-group difference in motor function and functional independence favouring telerehabilitation [21,33]. This suggests that telerehabilitation can positively influence patients’ functional independence and motor function, echoing findings from a previous systematic review [40]. However, while telerehabilitation may lead to significant improvements in functional independence and motor function, its superiority over conventional rehabilitation is not consistently demonstrated across all studies [42].

The limited follow-up in only a third of the included studies makes it difficult to draw conclusions regarding the sustainability of benefits from telerehabilitation interventions for adults with neurological conditions in LMICs, and therefore future studies should include a reasonable follow-up period in order to strengthen the evidence.

Despite its potential benefits, telerehabilitation also presents specific limitations that can impede broader adoption. One such challenge is the “digital divide,” where individuals or communities lacking reliable internet access, adequate devices, or sufficient technological literacy may be unable to participate fully in remote rehabilitation programmes [36]. In addition, maintaining data privacy and ensuring cybersecurity can be difficult in resource-constrained settings without robust regulatory frameworks and technological safeguards [43,44]. These issues can limit patient engagement and trust in telerehabilitation services and necessitate the need for investments in secure digital infrastructure in LICs contexts.

Acceptance and usability of telerehabilitation is key to its success in day-to-day practice. Telerehabilitation platforms must be perceived by patients and health professionals as user-friendly, dependable, secure, and able to facilitate efficient communication. Particularly in diverse LMICs contexts, patient engagement, adherence, and general satisfaction with telerehabilitation can be greatly increased by using user-friendly interfaces, accessible instructions, and culturally appropriate design [39,45,46]. However, the WHO has recommended that digital technologies, should not be viewed as a replacement of conventional therapy as both have unique advantages that can complement each other [8]. Therefore, an integrative model that combines both approaches could be a promising solution to rehabilitation challenges in LMICs.

Although this review was underpinned by published guidelines for conducting systematic review (PRISMA), there were limitations, notably the risk of publication bias as only published peer-reviewed studies were included in this review. The variation in participants, telerehabilitation interventions, comparators and outcomes presented considerable barriers to formulating definitive conclusions on the effectiveness of telerehabilitation in terms of interventions implemented.

There is a clear need for more comprehensive and methodologically robust research, particularly in low income countries, e.g., in Africa and South America that are currently underrepresented. This need aligns with the WHO’s acknowledgment of a global shortfall in healthcare professionals, particularly rehabilitation experts, a challenge most acute in LMICs [4]. These studies should aim to elucidate the specific challenges and opportunities unique to these contexts. Future research should also aim to ascertain the conditions under which telerehabilitation can serve as an effective intervention or a viable alternative to traditional rehabilitation approaches. The WHO Rehabilitation 2030 Call for Action highlights the imperative of integrating comprehensive rehabilitation services into global health systems to ensure equitable access and improved health outcomes [5]. Therefore, establishing this evidence base is critical for integrating telerehabilitation into healthcare strategies, research and policymaking, ultimately enhancing support for adults with neurological conditions in LMICs.

1 **Implications for practice**

Telerehabilitation offers a promising approach to delivering care in resource-limited environments, potentially overcoming barriers such as distance, transportation costs, and workforce shortages. Exercise-based interventions delivered via telerehabilitation were the most common in the literature reviewed and generally showed positive outcomes for adults with neurological conditions. In addition, Activities of daily living training, health education, counselling, diet modification and management of depression, delivered via telerehabilitation all showed positive outcomes for adults with a range of neurological conditions. Nevertheless, practitioners should consider the limitations of telerehabilitation in delivering hands-on therapeutic techniques essential for some people with neurological conditions. Where direct in-person contact (e.g., for hands-on treatment or real-time adjustments) are needed, scheduling periodic in-person sessions are important to maintain clinical efficacy. Additionally, practitioners must be aware of the digital divide and recognise that not all patients will have access to reliable internet or suitable devices [36], or know how to use these effectively. This will require screening for technological barriers and enablers before initiating telerehabilitation and, where possible, offering alternative or blended care models that combine in-person and telerehabilitation interventions. Also, continuing professional development in telerehabilitation for both clinicians and patients can further optimise engagement, reduce problems, and improve engagement with home-based rehabilitation.

2 **Implications for research**

Given the heterogeneity of the populations, interventions, outcomes, and measurement tools utilised in telerehabilitation research trials included in this review, there remains a need for more standardised research protocols to allow for more robust synthesis (e.g., meta-analyses). The lack of long-term follow-up data in most of the included studies limits the understanding of the sustained benefits of telerehabilitation interventions. Future studies should aim to incorporate longer follow-up periods and standardised outcomes to ascertain the durability of telerehabilitation benefits. Researchers might also explore rehabilitation domains beyond physical interventions to include cognitive, vocational, and communication interventions to provide a more holistic view of the potential of telerehabilitation.

Due to a paucity of data, subgroup analyses exploring how age, level of disability, socioeconomic status, or other variables may affect the effectiveness of telerehabilitation. Future studies should report this important information in order to address this knowledge gap. Addressing this may require collaboration among researchers, leveraging local expertise, and involving multidisciplinary teams skilled in technology, clinical practice, and community engagement. Furthermore, developing culturally tailored and context-specific interventions and assessment tools could further enhance the external validity of future research in LMICs.

3 **Implications for policy**

Policy initiatives are critical to addressing the infrastructural and legislative barriers that hinder telerehabilitation implementation. Lack of regulatory frameworks to protect data privacy, unstable power supplies and limited internet access remain significant challenges to telerehabilitation implementation. Strategic investments in broadband connectivity, energy infrastructure, and cybersecurity can help support service delivery and safeguard patient data. This may require collaboration with private-sector partners to fund and maintain telerehabilitation technologies, along with relevant workforce development programmes.

Clear guidelines on how telerehabilitation can complement existing in-person services is needed. The WHO Rehabilitation 2030 Call for Action [5] provides a blueprint for integrating rehabilitation services into mainstream health systems, but further customisation is needed to accommodate telerehabilitation’s unique requirements in LMICs. By embedding these services into broader health policy and allocating sustainable financing, governments can ensure that telerehabilitation becomes a viable and equitable part of the care continuum for adults with neurological conditions.

## 5. Conclusion

Our review indicates that telerehabilitation can meaningfully improve neurological outcomes in adults across several LMICs. However, evidence is almost entirely confined to short-term trials from middle-income settings, leaving two critical uncertainties: (i) whether benefits persist beyond a few months and (ii) whether the approach translates to LICs. Although telerehabilitation is widely promoted as a way to bypass workforce and geographic barriers in resource-limited health systems, we identified virtually no controlled trials from LICs, exposing a major evidence gap. To establish telerehabilitation as a sustainable, scalable strategy, the field now needs methodologically rigorous studies with longer follow-up, robust economic evaluations, and direct implementation in LIC contexts. Building this evidence base is essential not only to judge long-term effectiveness but also to inform policy, financing, and integration of telerehabilitation into routine rehabilitation services across the full spectrum of LMICs.

## Supporting information

S1 Appendix Search strategy. (DOCX)

S1 Checklist PRISMA checklist. (DOCX)
