# Supplementary material for: Effectiveness of telerehabilitation for adults with neurological conditions in low and middle income countries: A systematic review
Source: PLOS Digit Health. 2025 Jul 7;4(7):e0000911. doi: 10.1371/journal.pdig.0000911 (PMC12233225; doi:10.1371/journal.pdig.0000911)
Supplement: S1 Appendix — (DOCX) [file pdig.0000911.s001.docx]

S1 Appendix: SEARCH STRATEGY

| Tele-rehabilitation* |
| --- |
| Tele rehab*OR Tele-health OR Tele-medicine OR e-health OR mobile health OR mhealth OR Remote rehab* OR Virtual rehab* OR teleneurology |
| Neurological Conditions |
| Neurological disorder* OR Nervous system disorder* OR Nervous system disease* OR Stroke OR CVA OR Cerebrovascular Accident OR Spinal cord injur* OR SCI OR Parkinson disease, OR Multiple Sclerosis OR MS OR Cerebral palsy |
| Low- and middle-income |
| "Low and middle income" OR "low to middle income" OR "Third world" OR underdeveloped OR, less-developed OR "lower middle income" OR "lower-middle-income" OR Africa OR Asia OR "Developing country" OR LMIC OR Afghanistan OR Albania OR Algeria OR Angola OR Armenia OR Azerbaijan OR Bangladesh OR Belarus OR Belize OR Benin OR Bhutan OR Bolivia OR "Bosnia and Herzegovina" OR Botswana OR Brazil OR "Burkina Faso" OR Burundi OR "Cabo Verde" OR Cambodia OR Cameroon OR "Central African Republic" OR Chad OR China OR Colombia OR Comoros OR "Democratic Republic of Congo" OR Congo OR "Costa Rica" OR "Côte d'Ivoire" OR Cuba OR Djibouti OR Dominica OR "Dominican Republic" OR Ecuador OR Egypt OR "El Salvador" OR "Equatorial Guinea" OR Eritrea OR Eswatini OR Ethiopia OR Fiji OR Gabon OR Gambia OR Georgia OR Ghana OR Grenada OR Guatemala OR Guinea OR Guinea-Bissau OR Guyana OR Haiti OR Honduras OR India OR Indonesia OR Iran OR Iraq OR Jamaica OR Jordan OR Kazakhstan OR Kenya OR Kiribati OR "Democratic People's Republic of Korea" OR Kosovo OR Kyrgyzstan OR "Lao People's Democratic Republic" OR Lebanon OR Lesotho OR Liberia OR Libya OR "North Macedonia" OR Madagascar OR Malawi OR Malaysia OR Maldives OR Mali OR "Marshall Islands" OR Mauritania OR Mauritius OR Mexico OR Micronesia OR Moldova OR Mongolia OR Montenegro OR Montserrat OR Morocco OR Mozambique OR Myanmar OR Namibia OR Nauru OR Nepal OR Nicaragua OR Niger OR Nigeria OR Niue OR Pakistan OR Panama OR "Papua New Guinea" OR Paraguay OR Peru OR Philippines OR Rwanda OR "Saint Helena" OR Samoa OR "São Tomé and Príncipe" OR Senegal OR Serbia OR "Sierra Leone" OR "Solomon Islands" OR Somalia OR South Africa OR South Sudan OR Sri Lanka OR Saint Lucia OR "Saint Vincent and the Grenadines" OR Sudan OR Suriname OR "Syrian Arab Republic" OR Tajikistan OR Tanzania OR Thailand OR Timor-Leste OR Togo OR Tokelau OR Tonga OR Tunisia OR Turkey OR Turkmenistan OR Tuvalu OR Uganda OR Ukraine OR Uzbekistan OR Vanuatu OR Venezuela OR Vietnam OR "Wallis and Futuna" OR "West Bank and Gaza Strip" OR Yemen OR Zambia OR Zimbabwe |
